# Serum Nox1 and Gper Levels in Patients With Generalized Anxiety Disorder: A Cross‐Sectional Study in Turkiye

**DOI:** 10.1002/brb3.70645

**Published:** 2025-07-07

**Authors:** Onur Hurşitoğlu, Ergül Belge Kurutaş, Ömer Faruk Uygur, Nurinnisa Ozturk, Hilal Uygur, Fatih Saglam, Rebecca Strawbridge

**Affiliations:** ^1^ Department of Psychiatry, Faculty of Medicine Kahramanmaras Sutcu Imam University Kahramanmaras Turkey; ^2^ Department of Biochemistry, Faculty of Medicine Kahramanmaras Sutcu Imam University Kahramanmaras Turkey; ^3^ Department of Psychiatry, Faculty of Medicine Ataturk University Erzurum Turkey; ^4^ Department of Biochemistry, Faculty of Medicine Ataturk University Erzurum Turkey; ^5^ Department of Psychiatry Erzurum Training and Research Hospital Erzurum Turkey; ^6^ Department of Psychiatry Siirt Training and Research Hospital Siirt Turkey; ^7^ Centre for Affective Disorders, Department of Psychological Medicine, Institute of Psychiatry, Psychology and Neuroscience King's College London London UK

**Keywords:** biomarker, ERα, ERβ, estrogen, oxidative stress, reactive oxygen species

## Abstract

**Purpose:**

Although the etiopathogenesis of generalized anxiety disorder (GAD) is not fully known, it is suggested that oxidative stress (OS) and estrogen play important roles. NADPH oxidase 1 (NOX1) is from the NADPH oxidase (NOX) family, which is known as a main source of reactive oxygen species (ROS). The G protein‐coupled estrogen receptor (GPER) is a novel estrogen receptor that mediates the non‐genomic rapid effects of estrogen. Recent work has shown GPER to be associated with OS, but it has not to our knowledge been examined in patient samples with GAD.

**Method:**

We conducted a study comparing serum NOX1 and GPER levels of patients with GAD and healthy controls (HCs) matched for age, sex, and body mass index (BMI).

**Finding:**

We found increased NOX1 and decreased GPER levels in patients with GAD compared with HCs. In addition, we showed that serum NOX1 levels have very good diagnostic performance for patients with GAD. We observed a weak positive correlation between serum NOX1 levels and GPER levels in the patient group.

**Conclusion:**

Although we believe these results are important for a better understanding of the role of OS and estrogen in GAD patients, longitudinal studies should be performed in larger samples of GAD patients grouped by severity of symptoms.We investigated the levels of NOX1 and GPER in patients with GAD. We found increased NOX1 and decreased GPER levels in patients with GAD compared to healthy controls. In addition, we showed that serum NOX1 levels have very good diagnostic performance for patients with GAD.

## Graphical abstract

We investigated the levels of NOX1 and GPER in patients with Generalized Anxiety Disorder. We found increased NOX1 and decreased GPER levels in patients with GAD compared to healthy controls. In addition, we showed that serum NOX1 levels have very good diagnostic performance for patients with GAD.

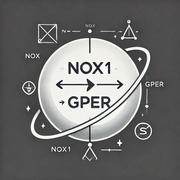

## 1 Introduction

GAD, with a lifetime prevalence of 5.7%, is characterized by excessive worry, fatigue, restlessness, and impaired concentration, irritability against daily stimuli, and can cause social, familial, and occupational disruptions (Kessler and Wang 2008, Fuller‐Thomson et al. 2022). Although it is a common illness in the community and an important cause of disability if not successfully treated, the etiology of GAD has still not been adequately clarified (Lebowitz et al. 2014). There is evidence that nitro‐oxidative stress plays an important role in the etiopathogenesis of anxiety disorders (Maes et al. 2018, Roomruangwong et al. 2018). In animal studies on nitro‐oxidative stress, an increase in nitrogen species (RNS) and ROS, and a decrease in antioxidant enzymes, have been reported to be associated with anxiety (Hassan et al. 2013, Malcon et al. 2020). Although short‐term increases in ROS have important physiological functions such as memory and learning, prolonged ROS levels that are significantly increased compared with antioxidant levels cause damage as a result of oxidation of cell components (Liu and He 2016, Radak et al. 2013).

Many enzyme activities have functions in the production of ROS, including cyclooxygenases, lipoxygenases, monooxygenases, respiratory chain enzymes, xanthine oxidase, and NADPH oxidase (NOX) (Goitre et al. 2012). The NOX enzyme family, which is the main source of ROS, has physiological roles in short‐term stress. However, it causes pathological changes in signal transmission pathways, migration, differentiation, proliferation, and cell death as a result of its excessive activation in long‐term stress (Brown and Griendling 2009, Teixeira et al. 2017). The NOX family consists of 7 homologs, NOX1‐5 and DUOX1‐2. NOX 1–5 has been observed in the central nervous system (CNS) (Lou et al. 2018, Hernandes et al. 2022). Recently, NOX1 has been shown to be associated with some brain and psychiatric disorders (Hursitoglu et al. 2023, Ma et al. 2017, Hurşitoğlu et al. 2022). To the best of our knowledge, no human GAD studies have yet examined serum levels of NOX1, whose main biological function is the production of superoxide by single electron reduction (Vermot et al. 2021).

There are three estrogen receptors: G protein‐coupled estrogen receptor (GPER), ERα, and ERβ. ERα and ERβ, also known as nuclear receptors, are responsible for slow genomic effects, while GPER mediates fast non‐genomic effects (Olde and Leeb‐Lundberg 2009). There is evidence for sex‐related differences between ERα and ERβ in behavioral responses to stress, and it has been suggested that ERβ mediates the anxiolytic effect (Imwalle et al. 2005, Walf et al. 2009). Recently, there has been an increased interest in animal studies on anxiety‐like behaviors about GPER and the GPER agonist G‐1. However, there are inconsistent results regarding whether GPER increases or decreases anxiety (Hart et al. 2014, Kastenberger et al. 2012, Kastenberger and Schwarzer 2014, Zheng et al. 2020). One study, by Fındıklı et al., reported increased serum GPER levels in GAD patients, and this pattern has also been observed in samples of people with schizophrenia and bipolar disorder (Fındıklı et al. 2016, Hursitoglu et al. 2020, Orhan et al. 2018).

It has been reported that estradiol, which is the most physiologically potent estrogen form, has important effects on the reproductive system, as well as inhibiting OS through rapid signaling and genomic pathways. Estradiol also increases antioxidant enzymes and inhibits angiotensin II‐induced NOX activation (Chen et al. 2008, Barton et al. 2019). Recently, it has been suggested that G36, as a GPER blocker, reduces ROS production by decreasing NOX1 levels, and that G36 may possess uses in OS‐related chronic diseases (Meyer and Barton 2018).

The above‐mentioned relationship inspired us to work together on serum NOX1 and GPER levels in patients with GAD, which we believe still needs to be examined for new biomarkers to better understand the illness etiology. In this study, we primarily aimed to compare serum NOX1 levels of patients with GAD and healthy controls (HCs) matched for age, sex and body mass index (BMI). To the best of our knowledge, there is no study on serum NOX1 levels in patients with GAD. Secondly, we aimed to compare serum GPER levels in both groups. In addition, we aimed to show the diagnostic value of NOX1 and the relationship of these markers with symptom severity.

## 2 Methods

Fifty‐four newly diagnosed GAD outpatients consented and participated in this study between 10.12.2022 and 10.03.2023. Ethical approval was obtained prior to recruitment initiation, by Erzurum Atatürk University, Faculty of Medicine Ethics Committee on 01.12.2022 (approval number: 18). Fifty‐five HCs matched for age, sex and BMI were also included in the study. A structured clinical interview (SCID) was administered to all participants by an experienced psychiatrist (First 2014). Written informed consent was obtained from the participants before inclusion in the study. Inclusion criteria for study participants were having no previous history of psychiatric illness, no previous or current psychotropic treatment, and no current major medical illness. In addition, the GAD group was required to meet the DSM‐5 criteria for GAD and not have any other comorbid psychiatric illness such as depressive disorders, other anxiety disorders, and obsessive‐compulsive disorder,, while the HC group were required to have no current (as well as past) psychiatric diagnoses. Exclusion criteria for study participants were use of hormone replacement therapy or antioxidant agents, smoking, alcohol or substance abuse, being in the postmenopausal and pregnancy period.

All participants provided sociodemographic data. An experienced psychiatrist recorded patients’ clinical features and applied the Hamilton Anxiety Scale (HAM‐A) to measure the severity of anxiety symptoms.

Venous blood samples were taken from the participants between 08:00 and 11:00 on a weekday after overnight fasting. Blood samples were then immediately transferred to the biochemistry laboratory. Serum was separated by centrifugation at 4000 rpm for 15 min in the laboratory. We kept the separated serum samples at –28°C until analysis. We measured serum NOX1 and GPER levels in the obtained serum samples using a quantitative enzyme immunoassay technique (ELISA) with a commercial kit (for NOX1: MyBioSource Company, USA; for GPER: SEG045Hu, Cloud‐Clone Corp., Houston, TX, USA).

### 2.1 Statistical Analysis

Continuous variables' distribution was considered normal where skewness and kurtosis values fell between –2 and + 2; all continuous variables were normally distributed. We present mean ± standard deviation and percentage values as descriptive statistics. Differences between general anxiety disorder and control groups were examined using independent sample t‐tests and a chi‐square test. We calculated the correlation between continuous variables (age, BMI, clinical features‐NOX1, and GPER) in the patient group using the Pearson correlation test. We conducted a ROC curve analysis to test the diagnostic performance of NOX1 and GPER in general anxiety disorder patients. We used the SPSS 20.0 program for all statistical analyses, and *p* < 0.05 was considered significant.

## 3 Results

This study enrolled 54 patients with GAD and 55 HCs with similar age (*p* = 0.879), sex (*p* = 0.912), BMI distribution (*p* = 0.105) and marital status (*p* = 0.437). Number of years of education was significantly higher in the control group (*p* = 0.001) (Table 1).

**TABLE 1:** Demographics and clinical characteristics of patients with GAD and healthy controls.

	GAD (*n* = 54)	Control (*n* = 55)	–*P*‐value
Age (years) mean ± *SD*)	32.41 ± 9.47	32.16 ± 7.08	0.879
Sex (male) (*n*: %)	23(43%)	24(44%)	0.912
BMI (kg/m^2^) (mean ± *SD*)	23.94 ± 3.30	24.92 ± 2.90	0.105
Education (years) (mean ± *SD*)	8.2 ± 3.17	12.1 ± 4.68	0.001
Married (versus single) (*n*: %)	42/12(78%)	40/15(73%)	0.437
Duration of illness (weeks) (mean ± *SD*)	48.09 ± 31.67		

*Note*: GAD: Generalized Anxiety Disorder, BMI: Body Mass Index.

**FIGURE 1:**
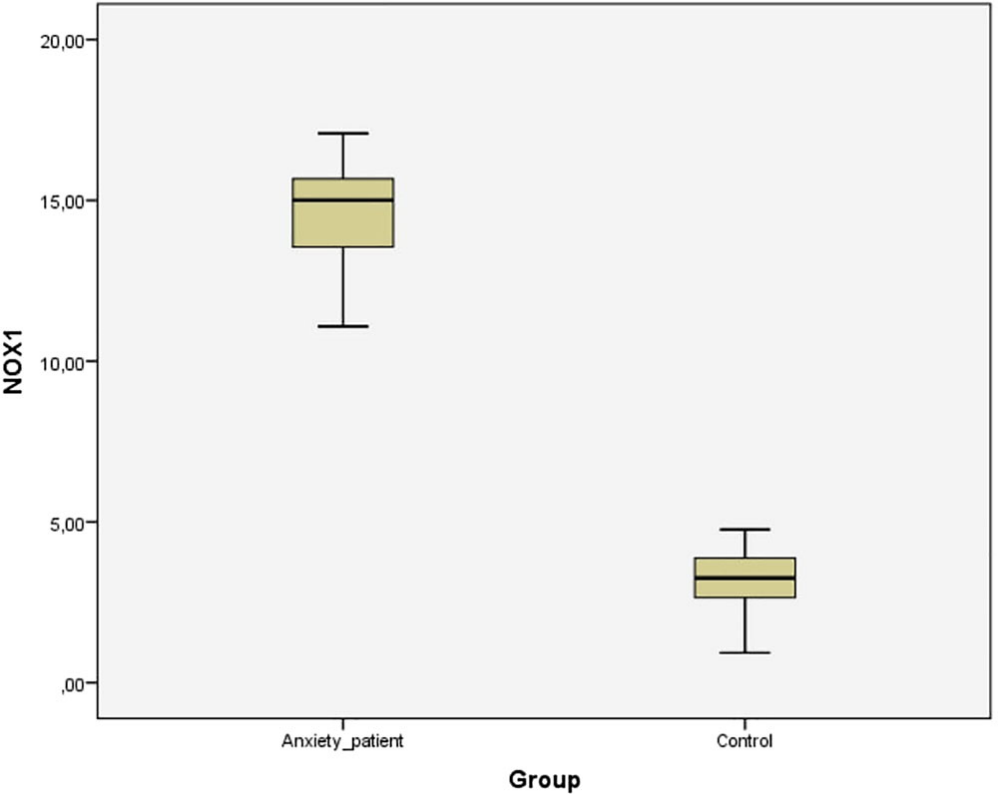
Boox plot graph for NADPH oxidase 1 (NOX1), representing highest, lowest, and mean values in both groups.

**FIGURE 2:**
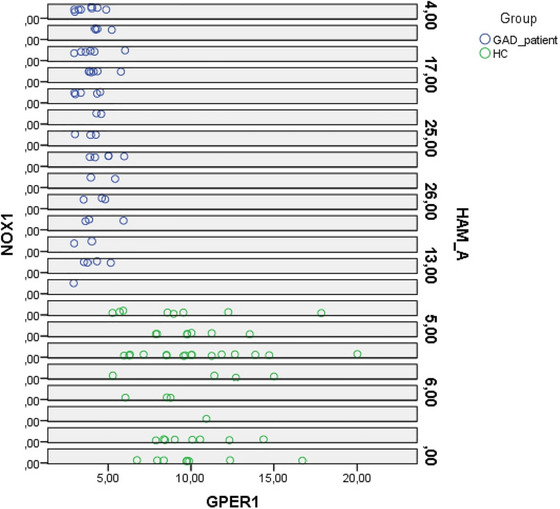
The relationship between NOX1‐HAM‐A and GPER‐HAM‐A in GAD patients and HCs.

The HAM‐A demonstrated on average a moderate severity (18.11 ± 3.99), significantly higher than HCs (2.76 ± 1.83) (*p* < 0.001). Serum NOX1 levels (14.59 ± 1.35) were significantly increased (*p* < 0.001), and GPER levels (4.06±0.69) were significantly decreased (*p* < 0.001) in patients with GAD compared to controls (Table 2) (Figure 1). Figure 2 displays the relationship between NOX1, GPER, and HAM‐A in the HC and GAD groups.

**TABLE 2:** Comparisons of questionnaire measures of anxiety and laboratory results of patients and healthy controls.

		GAD (*n* = 54)		Control (*n* = 55)	*P*‐value
HAM‐A	Mean ± *SD*	18.11 ± 3.99	2.76 ± 1.83		< 0.001
NOX1	Mean ± *SD*	14.59 ± 1.35	3.22 ± 0.82		< 0.001
GPER	Mean ± *SD*	4.06 ± 0.69	9.87 ± 2.77		< 0.001

*Note*: GAD: Generalized Anxiety Disorder, HAM‐A: The Hamilton Anxiety Rating Scale, NOX1: NADPH oxidaze1, GPER: G protein‐coupled estrogen receptor 1.

**FIGURE 3:**
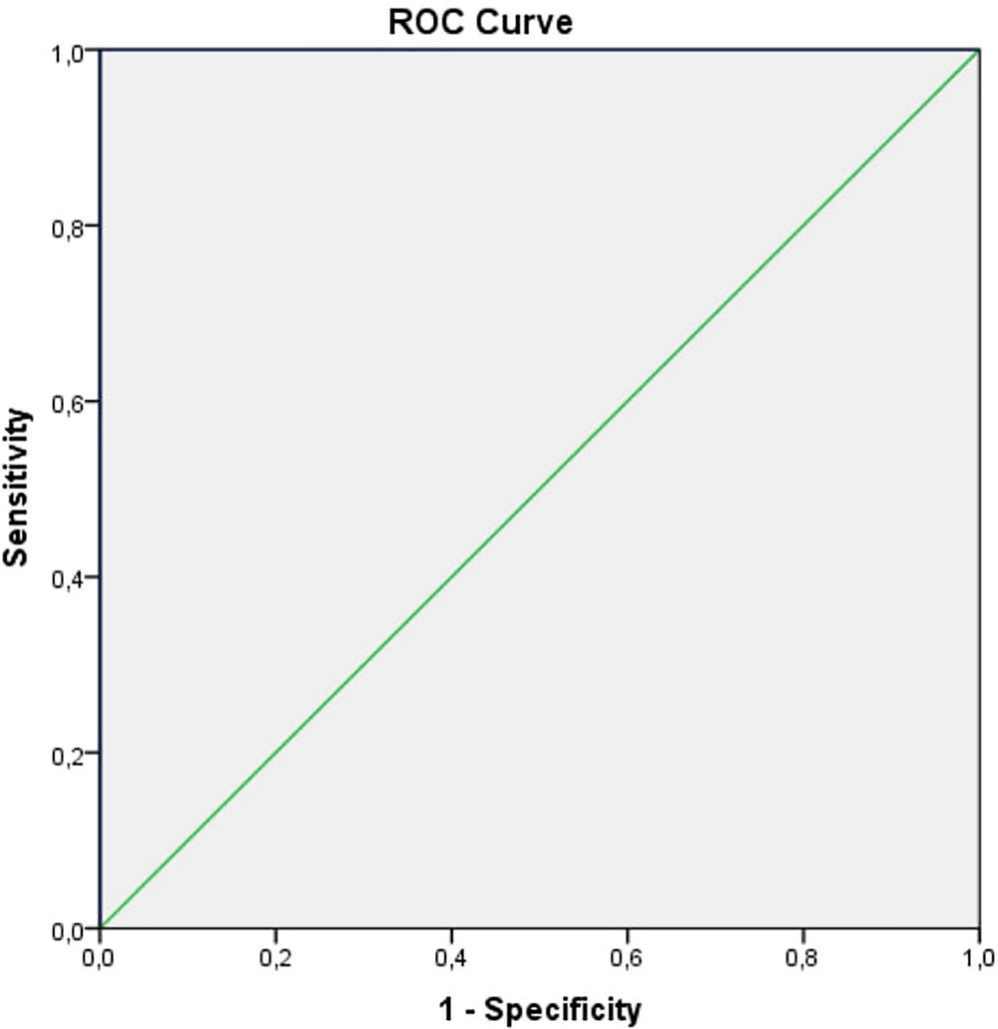
ROC curve for NOX1 (Sensitivity, 100%; Specificity, 98.2%; AUC was 1.00 for NOX1, The cut‐off point was detected as 5.45 ng/mL. NOX1 NADPH oxidase 1; AUC, area under the curve; ROC, receiver operating characteristic).

The lowest and highest bounds in patients with GAD for NOX1 (respectively 11.08 ng/ml 17.08 ng/ml) and controls (respectively 0.93 ng/ml, 4.76 ng/ml) are displayed in Figure 3. The ROC curve indicated NOX1 to have a very good diagnostic performance (AUC value = 1.00) (Figure 3).

We excluded the control group when evaluating the relationship between NOX1 and GPER levels and other variables. We did not find a significant relationship between symptom severity and NOX1 and GPER levels. We found a weak positive correlation between NOX1 and GPER levels (*p* < 0.05, *r* = 0.27). In addition, we found a moderate positive correlation between duration of illness and HAM‐A scores (*p* < 0.01, *r* = 0.50) (Table 3).

**TABLE 3:** Correlations between variables and GPER1, NOX1 levels in patient group.

Variables	Age	BMI	Illness duration	HAM‐A	NOX1	GPER
1.Age	—					
2.BMI	0.26	—				
3.Illness duration	0.02	0.04	—			
4.HAM‐A	−0.05	−0.06	0.50^**^	—		
5.NOX1	0.08	0.13	0.02	0.14	—	
6. GPER	−0.13	−0.18	0.04	0.00	0.27^*^	—

** *p* <0.01, ^*^ *p* < 0.05, HAM‐A: Hamilton anxiety rating scale, NOX1: NADPH oxidase1, GPER: G protein‐coupled estrogen receptor 1.

## 4 Discussion

To the best of our knowledge, the current study is the first to evaluate serum NOX1 levels (and the second study to evaluate serum GPER levels) in patients with GAD. In our study, we primarily found an increase in NOX1 levels, which is an important source of ROS, and a decrease in GPER levels, which mediates important functions of estradiol, in patients with GAD compared to HCs. Secondly, we found that serum NOX1 levels have very good diagnostic performance for patients with GAD. Thirdly, we observed a statistically significant weak positive correlation between serum NOX1 levels and GPER levels in the patient group. However, we did not find a significant relationship between serum NOX1 and GPER levels and symptom severity in patients with GAD.

There are recent studies investigating the role of NOX1 in psychiatric diseases and animal models of psychiatric diseases. Although there are important studies in the literature on the role of OS in anxiety, there are no studies on the role of NOX1 in patients with GAD (Maes et al. 2018, Hursitoglu et al. 2023, Hurşitoğlu et al. 2022, Ibi et al. 2017, Zhang et al. 2021, Asaoka et al. 2021). In studies examining the relationship between OS and anxiety observed in animal models, an increase in RNS, ROS, and malondialdehyde (MDA, an important lipid peroxidation marker) levels, and a decrease in antioxidant levels (e.g., glutathione peroxidase, catalase) have been reported (Hassan et al. 2013, Haider et al. 2015, Kumar and Chanana 2017, Hovatta et al. 2005, Rammal et al. 2008). In studies on lipid peroxidation markers in patients with GAD, significant and consistent results support that OS plays an important role in anxiety. Among these studies, Bulut et al. reported increased lipid hydroxy peroxidase levels and Findikli et al. increased MDA levels in the patient group, while in our recent study, we reported increased 8‐Iso‐Prostaglandin F2α levels in patients with GAD (Maes et al. 2018, Fındıklı et al. 2018, Hursitoglu O 2022). Despite the increase in oxidants, studies examining the role of antioxidants in patients with GAD have shown that antioxidant enzymes and specific antioxidants are decreased in patients compared to controls (Maes et al. 2018, Emhan et al. 2015, Kaya et al. 2013). In our study, we found that patients with GAD had increased NOX1 levels compared to HCs. In addition, NOX1 showed very good diagnostic performance in patients with GAD. We believe that these results we obtained regarding NOX1, which is known as an important source of ROS, support the deterioration of the oxidant‐antioxidant balance in favor of ROS and oxidants in the aforementioned studies. Although there have been no previous examinations of NOX1 in patients with GAD, there are relevant animal studies: Skurlova et al. reported increased NOX1 expression in the hippocampus of rats exhibiting anxiety‐like behaviors observed in the adjuvant arthritis process, and claimed that it plays an important role in the oxidative changes in the brain (Skurlova et al. 2011). Petrovic et al. found increased NOX2 levels in the amygdala in rats exposed to environmental stress and suggested that NOX could be a new potential target in post‐traumatic stress disorder and other anxiety disorders (Petrovic et al. 2018). When the increased NOX1 levels in patients with GAD are evaluated together with the NOX increases observed in rats in the aforementioned studies in the hippocampus and amygdala (important areas of the brain in anxiety), we believe that further studies should be conducted on the therapeutic effect of NOX downregulators in this group.

GPER, a new G protein‐coupled estrogen receptor known to be associated with many physiological events, has recently been shown to play a role in regulating estrogen's HPA axis and anxiety‐related functions (Zheng et al. 2020). Although there are data on the association of GPER with anxiety, mostly based on animal models, the data do not appear to be fully consistent. A study by Tien et al. reported that GPER agonists improved anxiety‐like behaviors in rats (Tian et al. 2013). Similarly, Zheng et al. recently reported an increase in anxiety‐like behaviors in ovariectomized rodents (OVX) and a reduction of these behaviors by the GPER agonist G‐1 (Zheng et al. 2020). However, it also has been reported that G‐1 either increases or does not alter anxiety levels in OVX mice (Hart et al. 2014, Kastenberger et al. 2012). In the only study conducted in humans, GPER levels were reported to be increased in patients with GAD compared to controls matched for sex and age (Fındıklı et al. 2016). In our study, we showed that patients with GAD had decreased GPER levels. In our study, the control group was matched by BMI as well as age and sex. Davis et al. reported that GPER regulates body weight as well as body fat (Davis et al. 2014). For this reason, we believe that the result of decreased GPER levels in the patient group we obtained in our study is among the groups with sufficient homogeneity and is reliable for potential biomarker examination. In addition, while symptom severity was mild (HAM‐A scores: 13.80 ± 2.1) in the previous study, patients in our study had moderate (HAM‐A scores: 18.11 ± 3.99) symptom severity. However, no correlation was found between GPER levels and either symptom severity or BMI in our study. Inconsistent results between our studies may be partially explained by symptom severity (i.e., not decreased in milder GAD), and BMI (matched groups and low intra‐sample variability in our study). When the animal studies (GPER, G‐1) and human patient studies (GPER) are evaluated together, the findings may suggest that G‐1 could be useful in the treatment of anxiety patients with only moderate and severe symptoms.

Recent studies suggest that the regulatory effects of estrogen on mitochondrial and OS may be mediated by GPER (Frick et al. 2015, Kumar et al. 2015). Wang et al. reported that estrogen deficiency caused disruption of mitochondrial membrane potential and antioxidants in rats, whereas the GPER agonist G‐1 improved superoxide dismutase, mitochondrial membrane potential, and total antioxidant capacity (Wang et al. 2021). Although serious beneficial effects mediated by GPER agonists are known, Meyer and Barton 2018. reported associations between GPER and NOX1 but not the other key NOX isoforms (NOX2 or NOX4). They also found that G36, a GPER blocker, reduced NOX1 expression only (Meyer and Barton 2018). To the best of our knowledge, the current study is the first to examine serum NOX1 and GPER levels together in humans, and we detected increased NOX1 and decreased GPER levels in the patient group. Increased NOX1 levels and decreased GPER levels in the patient group seem to contradict Meyer and Barton 2018 findings (that a GPER blocker downregulates NOX1). However, we believe that there may be many mechanisms that could cause increased NOX1 and decreased GPER levels in the patient group rather than the relationship between them. But for all that, the positive weak correlation between GPER and NOX1 levels we obtained may support Meyer and Barton 2018 results that GPER deficiency causes a decrease in NOX1.We recommend that longitudinal studies in patients with GAD (stratified by severity) could better elucidate the relationship between GPER and OS in patients with GAD and ultimately may offer new therapeutic agents.

Although we believe that our application of robust inclusion criteria and blood draw protocol in the drug‐naive non‐smoking patient group and age‐sex BMI matched HCs makes it a powerful study evaluating potential biomarkers, it has some important limitations. First of all, our study had a small sample size and should be supported by larger studies. Second, since it is a cross‐sectional study, it is insufficient to show the causal relationship of these parameters. Third, although it may seem advantageous because it is easy to take samples from the serum and many molecules can pass through the blood‐brain barrier, it may not be considered that the values we obtain are a direct reflection of the processes in the brain. Fourth, since the biochemical processes are quite complex and related to many factors, the study we examined with two biomarkers is insufficient to provide information about the general process.

In conclusion, we found increased NOX1 and decreased GPER levels in patients with GAD compared to HCs matched for age, sex and BMI. In addition, we showed that serum NOX1 levels have very good diagnostic performance for patients with GAD. Finally, we observed a weak positive correlation between serum NOX1 levels and GPER levels in the patient group. Although we believe that these results are important for a better understanding of the role of OS and estrogen in patients with GAD, it should be considered a preliminary study, and further studies on the subject are required.

## Author Contributions

**Onur Hurşitoğlu**: Methodology, investigation, writing ‐ original draft. **Ergül Belge Kurutaş**: Investigation, writing ‐ review and editing, supervision, formal analysis. **Ömer Faruk Uygur**: Writing ‐ review and editing, investigation. **Nurinnisa Ozturk**: Formal analysis, writing ‐ review and editing. **Hilal Uygur**: Writing ‐ review and editing, investigation. **Fatih Saglam**: Investigation, writing ‐ review and editing. Rebecca Strawbridge: writing ‐ review and editing, supervision.

## Conflicts of Interest

The authors declare no conflicts of interest.

## Peer Review

The peer review history for this article is available at https://publons.com/publon/10.1002/brb3.70645

## Data Availability

The data that support the findings of this study are available on request from the corresponding author, [O, H].
